# A time-course analysis of *Aspergillus terreus* secretomes reveals the importance of pectin-degrading enzymes to increase the digestibility of soybean meal

**DOI:** 10.1128/aem.02153-23

**Published:** 2024-08-20

**Authors:** Lauriane Plouhinec, Estelle Bonnin, Mélodie Kielbasa, Jean Armengaud, Virginie Neugnot, Jean-Guy Berrin, Mickael Lafond

**Affiliations:** 1 INRAE, Aix-Marseille Université, UMR 1163 Biodiversité et Biotechnologie Fongiques, Marseille, France; 2 Adisseo France S.A.S, CINAbio, INSA Toulouse, Toulouse, France; 3 INRAE, Université de Nantes, UR 1268 Biopolymères Interactions Assemblage, Nantes, France; 4 Département Médicaments et Technologies pour la Santé (DMTS), Université Paris-Saclay, CEA, INRAE, Bagnols sur Cèze, France; Anses, Maisons-Alfort Laboratory for Food Safety, Maisons-Alfort, France

**Keywords:** carbohydrate-active enzymes, filamentous fungi, soybean meal, pectins, rhamnogalacturonan-I, animal feed

## Abstract

Considering an ever-growing global population, which hit 8 billion people in the fall of 2022, it is essential to find solutions to avoid croplands competition between human food and animal feed. Agricultural co-products such as soybean meals have become important components of the circular economy thanks to their use in animal feed. Their implementation was made possible by the addition of exogenous enzymes in the diet of monogastric animals, especially fungal carbohydrate-active enzymes (CAZymes). Here, we describe a time-course production and analysis of *Aspergillus terreus* secretomes for the identification of CAZymes able to enhance the digestibility of soybean meals. Functional assays revealed that the release of nutrients and the degradation of pectins in soybean meals can be tightly interconnected. Using a comparative proteomics approach, we identified several fungal pectin-degrading enzymes leading to increased assimilable nutrients in the soluble fraction of soybean meals. Our results reinforce the importance of deconstructing pectic polysaccharides in feedstuffs and contribute to sharpen our understanding of the fungal enzymatic interplays involved in pectin hydrolysis.

**IMPORTANCE:**

In the present study, we developed a strategy to identify the key fungal enzymatic activities involved in the improvement of soybean meal (SBM) digestibility. Our data unravel the importance of pectin degradation for the release of nutrients from SBM and provide some insights regarding the degradation of rhamnogalacturonan-I (RG-I) by ascomycetes. Indeed, the hydrolysis of pectins and RG-I by human microbiota is well documented in the literature, but our knowledge of the fungal CAZymes at play for the degradation of soybean pectins remains hitherto underexplored. Due to its wide use in animal feed, improving the digestibility of SBM by enzymatic treatments is a current challenge for feed additive suppliers. Since non-starch polysaccharides and pectins have often been reported for their anti-nutritional role in SBM, we believe this study will provide new avenues toward the improvement of enzymatic cocktails for animal nutrition and health.

## INTRODUCTION

With a global population forecasted at 10 billion people in 2050 and the depletion of our planet’s resources, competition between human food and animal feed is arising (1, 2). The challenge of feeding the human population and animals properly will become increasingly important in the coming decades, which is why the animal feed industry seeks alternative feeding solutions. The implementation of agricultural co-products such as soybean meal (SBM) in the formulation of poultry diets is one example of such alternative solution (3). SBM’s high protein contents and low production costs are interesting assets to partially replace exogenous amino acids in the diet of monogastric animals (4). However, the addition of SBM to animal meals also increases its indigestibility due to the richness of SBM in non-starch polysaccharides (NSPs) (5). While the cereal base part of animal feed presents high cellulose and hemicellulose contents, pectins are reported to be the main NSPs in oilseeds (6).

Their heterogeneity and wide diversity make pectins the most complex polysaccharides in the plant kingdom. Indeed, pectins can be composed of at least 17 different monosaccharides arranged together in as many different linkages and structures (7). The “smooth” part of pectins, called homogalacturonan, is a polymer of α-(1,4)-linked d-galacturonic acid (d-Gal*A*) residues partly esterified with methyl and acetyl groups. In contrast, rhamnogalacturonan-I (RG-I) is often referred to as the “hairy” region of pectins. Its backbone is composed of d-Gal*A* and l-rhamnose (l-Rha) residues alternatively linked in α-(1,4) and α-(1,2). l-Rha residues can be branched by arabinans, galactans, and arabinogalactans, whose terminations could be decorated by a d-glucuronic acid (d-Glc*A*) or an l-fucose residue (8, 9). Furthermore, RG-I side chains can be linked to each other through di-ferulic bridges (10). Thus, multiple enzymatic activities are required to fully degrade RG-I (11). The deconstruction of RG-I facilitates its assimilation and, by extension, enhances the nutritional potential of SBM. In addition to the SBM protein bodies being entrapped in a complex polysaccharidic matrix (12), it is known that most of the RG-I in plant cell walls is covalently linked to arabinogalactan proteins (13). Therefore, RG-I can be one main obstacle to the correct assimilation of SBM nutrients.

Being natural plant degraders in the environment, filamentous fungi possess a wide enzymatic portfolio with many interesting candidates for the deconstruction of NSPs in soybean meal (14, 15). Nowadays, many fungal enzymatic cocktails containing carbohydrate-active enzymes (CAZymes) are available on the animal feed market (16). One of the most often used is Rovabio Advance, produced by the fermentation of *Talaromyces versatilis* and commercialized by Adisseo (17). The activity of Rovabio cocktails on cereals has been deeply characterized *in vitro* and *in vivo* (18–21), but only scarce information is available on its enzymatic efficiency against SBM. By analyzing the sugar content of SBM residual dry matter, we recently showed that the addition of *Aspergillus terreus* secretomes to Rovabio Advance significantly reduced the amount of pectin monomers in the residue (22). Uronic acids (UAs), fucose, arabinose, and galactose amounts were decreased by 1.4-, 1.5-, 1.9-, and 2.2-fold, respectively, suggesting an improvement of SBM pectin degradation in the presence of *A. terreus* secretomes. It is well known that filamentous fungi can adapt to the carbon source they are facing by dynamically mobilizing the set of enzymes they secrete to break down and metabolize recalcitrant polysaccharides during the degradation process (23–26). In this study, we undertook a time-course analysis of *A. terreus* secretomes using sugar beet pulp (SBP) and banana peels as culture inducers to favor the secretion of pectin-degrading CAZymes. We followed the degradation of SBM and soybean RG-I by fungal secretomes, analyzing in-depth the soluble products released. Matrix-driven correlation of fungal secretomes analyzed using proteomics and functional assays carried out on SBM and RG-I led to the identification of a set of fungal CAZymes playing a role in the hydrolysis of SBM pectins to enhance the digestibility of SBM.

## RESULTS

### Protein secretion by *A. terreus* is modulated by time and inducer of culture

The sugar composition of SBP and banana peels revealed high amounts of UAs, exhibiting the presence of pectins. Although the rhamnose content was below the detection limit in banana peels, significant amounts of arabinose and galactose were found in both culture inducers, suggesting the presence of RG-I (Table S1). The quantification of total *A. terreus* proteins in the supernatant demonstrated an increase of proteins secreted in the culture over time (Fig. S1A), and globally, more proteins were detected in the cultures on SBP compared to banana peels. The SDS-PAGE profile of fungal secretomes disclosed differences in their protein composition, depending on the time of culture and the inducer used (Fig. S1B). While some proteins appeared to be constitutively secreted by *A. terreus* whatever the culture conditions, some of them were specifically secreted on SBP. Furthermore, the electrophoretic analysis exposed a dynamic evolution of the secretomes’ protein composition over time, with some proteins appearing only at day 5 (D5) of culture and others fading away after day 3 (D3) of culture. At this stage, as the biological replicates displayed similar protein profiles and concentrations and due to the number of functional assays planned in this study, we pooled the culture supernatant duplicates together.

### *A. terreus* secretomes increase the digestibility of soybean meal

**Fig 1:**
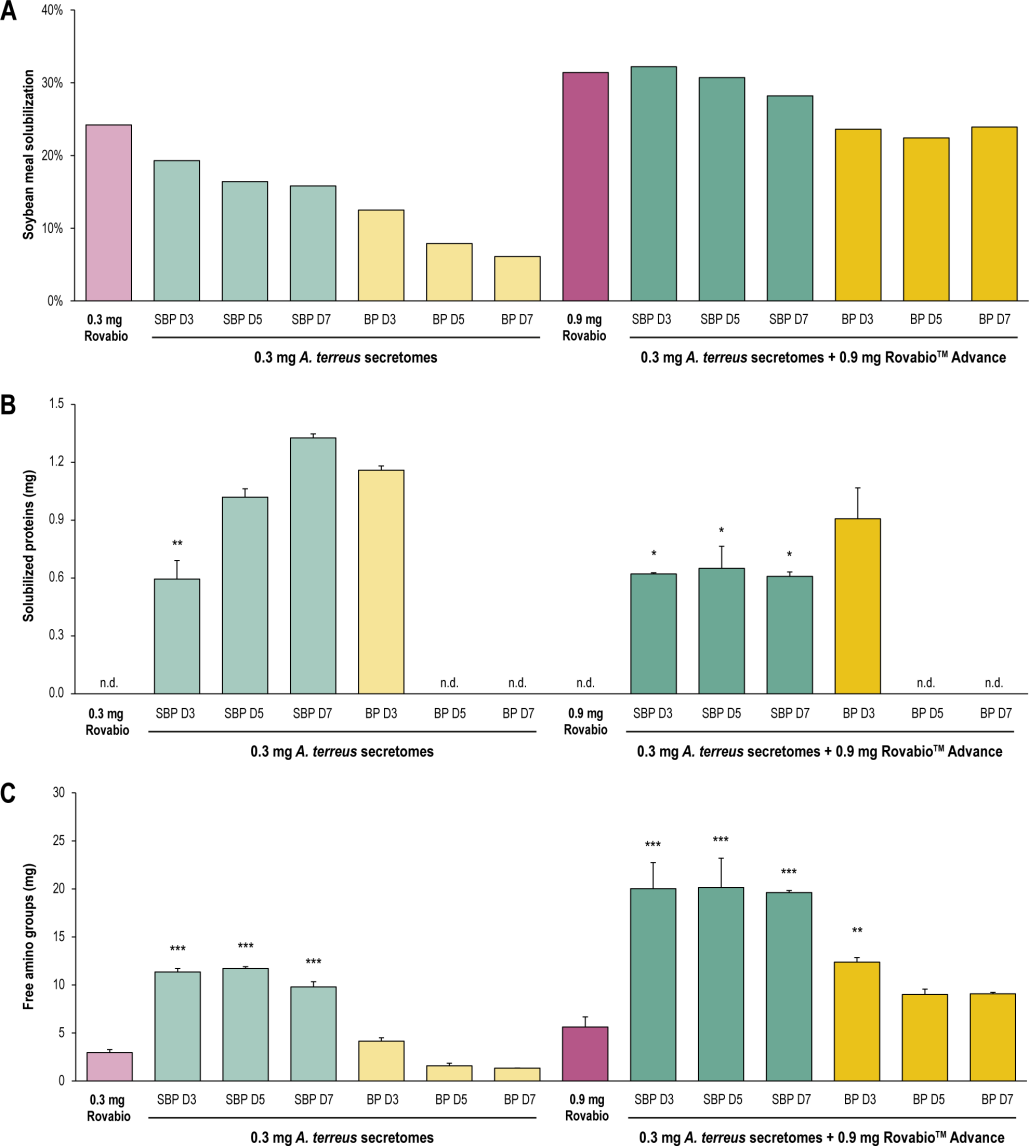
*In vitro* digestibility of SBM after 48-h hydrolysis by fungal secretomes. (**A**) Percentage of SBM residual matter. (**B**) Quantification of total proteins in the soluble fraction by the Bradford assay. (**C**) Quantification of free amino groups in the soluble fraction by the TNBS assay. All values are corrected by the control without enzyme and the control without substrate. Error bars correspond to standard deviations calculated from technical replicates (*n* = 2). Statistical analyses were performed using analysis of variance and post hoc Tukey’s test. *P < 0.05, **P < 0.01, ***P < 0.001. Hydrolysis tests were conducted at 37°C for 48 h. The inducers (SBP and BP) and the time of secretome harvest (D3, D5, and D7) are indicated. BP, banana peel; D7, day 7; n.d., not detected; SBP, sugar beet pulp.

To evaluate the impact of *A. terreus* secretomes on the *in vitro* digestibility of SBM, the solubilization of SBM was evaluated using three different markers (Fig. 1). Effects on the release of SBM proteins, peptides, and amino acids in the soluble fraction were assessed both by the quantification of total proteins and free amino groups using Bradford and 2,4,6-trinitrobenzene sulfonic acid (TNBS) assays, respectively. Evaluation of the digestibility of SBM was calculated based on the percentage of SBM solubilization after hydrolysis, i.e., corresponding to the decrease in the insoluble fraction, as previously described (22). Using a time-course analysis of SBM degradation by the commercial cocktail Rovabio Advance (Fig. S2), we set the time of reaction at 48 h of hydrolysis. The enzyme doses used for the *A. terreus* secretomes and for the Rovabio Advance cocktail were set according to our previous study, namely, 0.3 and 0.9 mg of total proteins, respectively (22).

After 48-h hydrolysis, the *A. terreus* secretomes used alone (0.3-mg enzyme load) solubilized SBM up to 19%, depending on the inducer used and the day of secretome harvest (Fig. 1A). Comparative analyses revealed that the secretomes obtained on SBP were more efficient than those obtained on banana peels. Moreover, the secretomes collected at D3 of culture were more efficient to solubilize SBM than the ones collected at D5 and day 7 (D7) for both culture inducers. At the same enzyme load, *A. terreus* secretome produced at D3 on SBP and Rovabio Advance could equally solubilize SBM. In these conditions, no significant enhancement of SBM solubilization was observed when adding the fungal secretomes to Rovabio Advance. However, the quantification of total proteins (Fig. 1B) and of free amino groups (Fig. 1C) in the soluble fraction of SBM exhibited significant differences between *A. terreus* secretomes alone and Rovabio Advance alone. Strikingly, *A. terreus* secretomes could release up to 1.3 mg of proteins after 48 h of hydrolysis (Fig. 1B). Interestingly, the increase of protein solubilization by fungal secretomes was correlated with the time of culture on SBP. However, the secretomes produced on banana peels behaved differently, as only the secretome collected at D3 could solubilize proteins from SBM after 48 h. Furthermore, the quantification of free amino groups in the soluble fraction of SBM confirmed that all fungal secretomes produced on SBP could outperform Rovabio Advance at 0.3-mg enzyme load (Fig. 1C).

The supplementation of Rovabio Advance by *A. terreus* secretomes significantly increased the solubilization of SBM proteins, up to 0.9 mg with the secretome produced at D3 on banana peels (Fig. 1B). Surprisingly, the secretomes produced on SBP at D5 and D7, as well as the one produced on banana peels at D3, are able to solubilize more proteins when used alone than when used in addition to the Rovabio Advance (*P* value of <0.05), suggesting the proteins are further hydrolyzed in the presence of Rovabio Advance. This hypothesis was confirmed by a considerable increase of free amino groups in the soluble fraction of SBM, from 5.6 mg with Rovabio Advance alone up to 20 mg when supplementing it with *A. terreus* secretomes produced on SBP (Fig. 1C).

Altogether, these results reveal that supplementing Rovabio Advance with *A. terreus* secretomes significantly enhances the digestibility of SBM by releasing higher amounts of peptides and amino acids by up to fourfold, although the insoluble residual matter of SBM is not significantly reduced. This observation could be explained by a better degradation of soluble NSPs found in SBM in the presence of fungal secretomes, potentiating access to the protein fraction of SBM for proteases and peptidases secreted by *A. terreus* and supplied by Rovabio Advance. Consequently, based (i) on this hypothesis, (ii) on the fact that the most abundant soluble NSPs in SBM are pectic polysaccharides, and (iii) on our previous demonstration of an activity of *A. terreus* secretomes on pectins (22), we undertook a detailed study of the enzymatic mechanisms involved in SBM pectin degradation by the fungal secretomes.

### SBM’s pectin backbone degradation by Rovabio Advance is improved in the presence of *A. terreus* secretomes

To evaluate the degradation of pectins by *A. terreus* secretomes, we followed the release of several monomeric units that make up pectins: UAs, rhamnose, arabinose/galactose, and fucose. While UA and rhamnose are markers of the hydrolysis of pectin backbone, arabinose, galactose, and fucose are directly related to the hydrolysis of pectin side chains (Fig. S3). The strategy of selecting several hydrolysis markers can be compared to a fingerprinting of SBM pectin hydrolysis in the different conditions tested. Of note, the release of arabinose, galactose, and fucose can also indicate the hydrolysis of the hemicelluloses (e.g., arabinoxylan or galactomannan) from SBM (27, 28). However, rhamnose and galacturonic acid (Gal*A*) can only be released from pectins (9, 29, 30).

**Fig 2:**
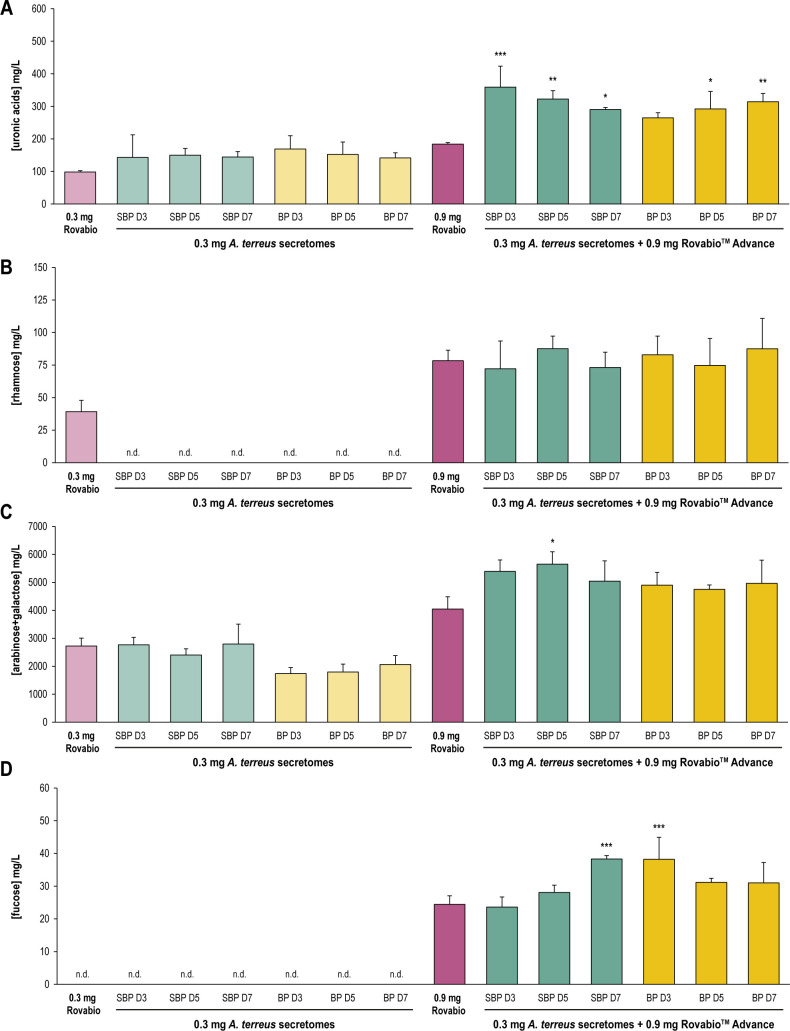
Quantification of pectin monomers in the soluble fraction of SBM after 48-h hydrolysis. (**A**) Uronic acids. (**B**) Rhamnose. (**C**) Arabinose + galactose. (**D**) Fucose. All values are corrected by the control without enzyme and the control without substrate. Error bars correspond to standard deviations calculated from technical replicates (*n* = 3). Statistical analyses were performed using analysis of variance and post hoc Tukey’s test. *P < 0.05, **P < 0.01, ***P < 0.001. Hydrolysis tests were conducted at 37°C for 48 h. The inducers (SBP and BP) and the time of secretome harvest (D3, D5, and D7) are indicated. BP, banana peel; n.d., not detected; SBP, sugar beet pulp.

Interestingly, at 0.3-mg enzyme load, *A. terreus* secretomes appeared to be as efficient as the commercial cocktail Rovabio Advance for the hydrolysis of uronic acids from SBM (Fig. 2A). Further analysis of the soluble fraction by high-performance anion-exchange chromatography coupled with pulsed amperometric detection (HPAEC-PAD) confirmed that more than 90% of UA quantified by the Megazyme enzymatic kit corresponds to galacturonic acid (Fig. S4), meaning the fungal secretomes and the Rovabio Advance can both degrade the backbone of SBM pectins. Conversely, none of the fungal secretomes gave better results than Rovabio Advance for the release of arabinose and galactose from SBM (Fig. 2C). At 0.3-mg enzyme load, Rovabio Advance also outperformed *A. terreus* secretomes for the release of rhamnose from SBM (Fig. 2B). Regarding fucose, neither the *A. terreus* secretomes nor the Rovabio Advance released a detectable amount of fucose (0.3-mg enzyme load, Fig. 2D). These results were not surprising, considering the scarce amount of fucose found in SBM (Table S1). Noteworthy, the assays used for the quantification of pectins degradation could only detect sugar monomers; therefore, we have no information on pectin oligosaccharides that could be present in the soluble fraction of SBM. Mass spectrometry analysis of the samples could provide more information on the oligosaccharides released during partial degradation of pectin by secretomes such as homogalacturonan oligosaccharides.

Furthermore, a clear effect of Rovabio Advance supplementation by *A. terreus* secretomes was observed on the release of UA from SBM (Fig. 2A). Surprisingly, while secretome efficiency for UA hydrolysis decreased over time of cultivation on SBP, it increased on banana peels, suggesting a different secretion pattern of the enzymes at play. We observed a higher release of UA in the Rovabio Advance condition supplemented with SBP D3 secretome (*P* value of < 0.001), suggesting an early-stage secretion of pectinases by *A. terreus* upon growth on SBP. No significant effect was observed on rhamnose release when Rovabio Advance was supplemented by *A. terreus* secretomes (Fig. 2B), which is consistent with the results obtained for *A. terreus* secretomes tested alone. Conversely, the supplementation with SBP D5 secretome induced a significant increase of arabinose and galactose release (*P* value of <0.05), indicating an additional effect of this secretome on Rovabio Advance for the degradation of SBM hemicelluloses and pectin side chains (Fig. 2C). Moreover, a significant interplay was observed between Rovabio Advance and the secretome produced on SBP at D7 for the release of fucose (*P* value of <0.001), with an increase from 24 to 38 mg/L (Fig. 2D). The same observation was made with the secretome produced on banana peels at D3 (*P* value of <0.001), which is all the more remarkable as none of the secretomes used alone were able to hydrolyze fucose from SBM.

Although neither fucose nor rhamnose was detected in the ethanol insoluble matter of SBM (Table S1), previous compositional analysis of soluble soybean polysaccharides displayed significant amounts of rhamnose, confirming the presence of RG-I (31, 32). Based on this knowledge, *A. terreus* secretome efficiency to hydrolyze SBM pectins was further characterized on soybean RG-I.

### *A. terreus* secretomes enhance the degradation of soybean RG-I side-chain ends by Rovabio Advance

With RG-I being the main component of pectins found in oilseeds, we studied the efficiency of *A. terreus* secretome on commercial soybean RG-I. According to the supplier data, the soybean RG-I we used contains 51% galacturonic acid for 49% neutral sugars (wt/wt). Neutral sugar content consists of 13.7% xylose, 12.3% galactose, 10.3% fucose, 6.4% rhamnose, 3.4% arabinose, and 1.5% other sugars (wt/wt). The contents of fucose and xylose relative to rhamnose are surprising, if we consider the current knowledge of RG-I composition, where xylose and fucose were found to represent traces of neutral sugar content (8, 13, 33). Moreover, the molar ratio of galacturonic acid:rhamnose is above previously reported ratios for soybean polysaccharides (32), which could indicate a contamination by homogalacturonan and/or xylogalacturonan (27).

**Fig 3:**
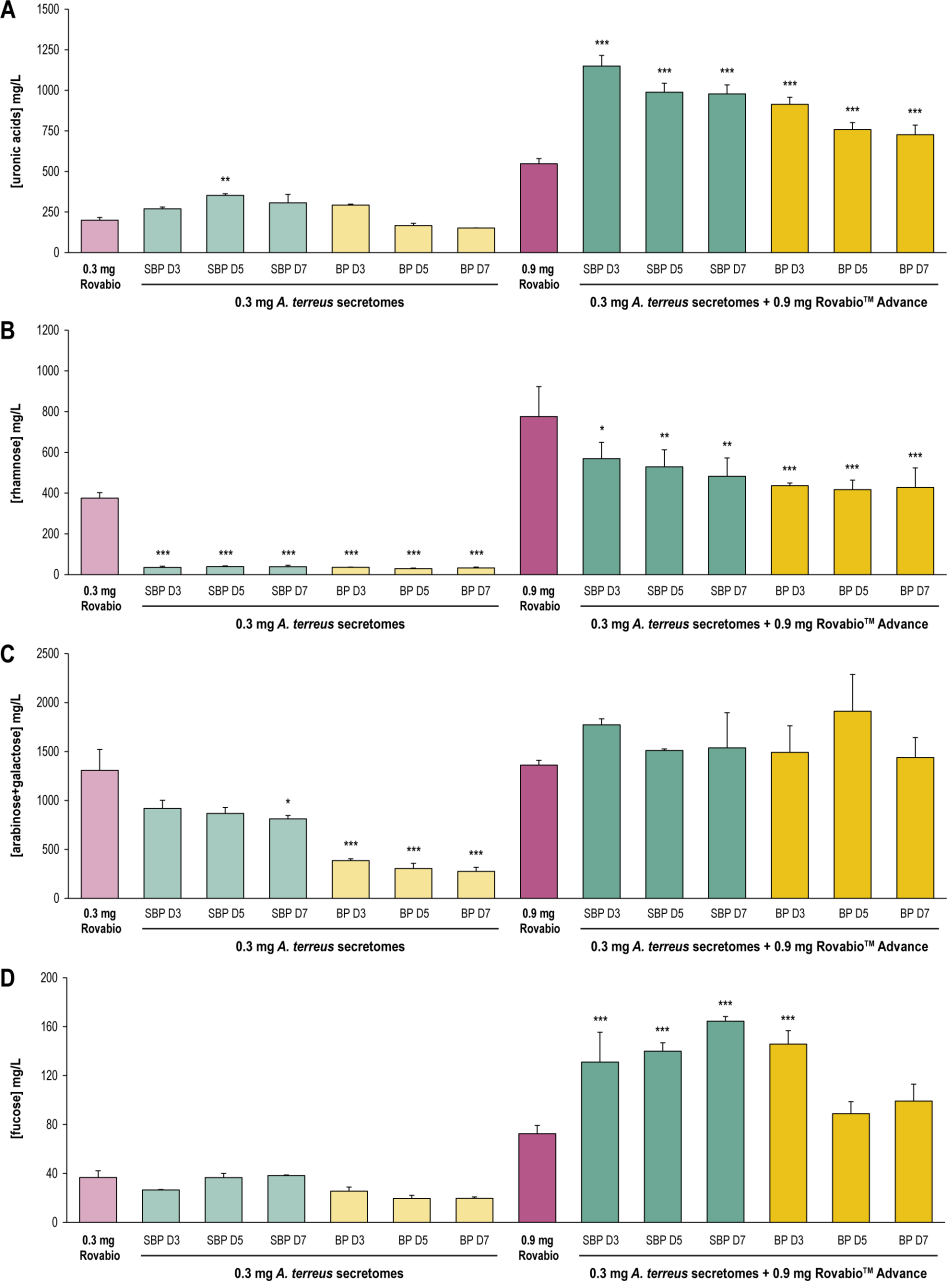
Quantification of pectin monomers in the soluble fraction of soybean RG-I after 48-h hydrolysis. (**A**) Uronic acids. (**B**) Rhamnose. (**C**) Arabinose + galactose. (**D**) Fucose. All values are corrected by the control without enzyme and the control without substrate. Error bars correspond to standard deviations calculated from technical replicates (*n* = 3). Statistical analyses were performed using analysis of variance and post hoc Tukey’s test. *P < 0.05, **P < 0.01, ***P < 0.001. Hydrolysis tests were conducted at 37°C for 48 h. The inducers (SBP and BP) and the time of secretome harvest (D3, D5, and D7) are indicated. BP, banana peel; SBP, sugar beet pulp.

Overall, the concentrations of UA, rhamnose, and fucose measured in soybean RG-I soluble fraction after hydrolysis (Fig. 3) were much higher than the concentrations measured in SBM soluble fraction (Fig. 2). Conversely, more arabinose and galactose were detected after SBM hydrolysis than after soybean RG-I hydrolysis due to the presence of hemicelluloses in SBM. Interestingly, supplementing the Rovabio Advance with *A. terreus* secretomes showed an interesting synergistic effect for the hydrolysis of UA and fucose from soybean RG-I. Fold changes for UA release between Rovabio Advance alone and Rovabio Advance supplemented by secretomes are quite similar during SBM and RG-I degradation (Fig. 2A and 3A). Rovabio Advance supplemented by the secretome produced on SBP at D7 released up to 2.8 more fucose than Rovabio alone (Fig. 3D, *P* value of <0.001). While the supplementation did not significantly increase the release of arabinose and galactose from RG-I (Fig. 3C), it seems the addition of *A. terreus* secretomes to Rovabio Advance had a negative impact on rhamnose hydrolysis from RG-I (Fig. 3B, *P* value of <0.01), as the concentration of rhamnose in the soluble fraction decreased from 775 to around 430 mg/L with secretomes produced on banana peels. These results are not consistent with our observations on SBM, suggesting that the presence of structural differences between soybean meal RG-I and commercial soybean RG-I had an impact on the efficacy of the RG hydrolases and rhamnosidases secreted by *A. terreus*. The calculation of RG-I hydrolysis percentages showed that none of the conditions tested resulted in complete hydrolysis of RG-I after 48 h (Table S2), indicating that some specific motifs hamper the action of pectin-degrading enzymes present in Rovabio Advance and/or in *A. terreus* secretomes.

### Shotgun proteomics reveal polarized secretion profiles of pectin-degrading enzymes by *A. terreus*, depending on time of culture and inducers

**Fig 4:**
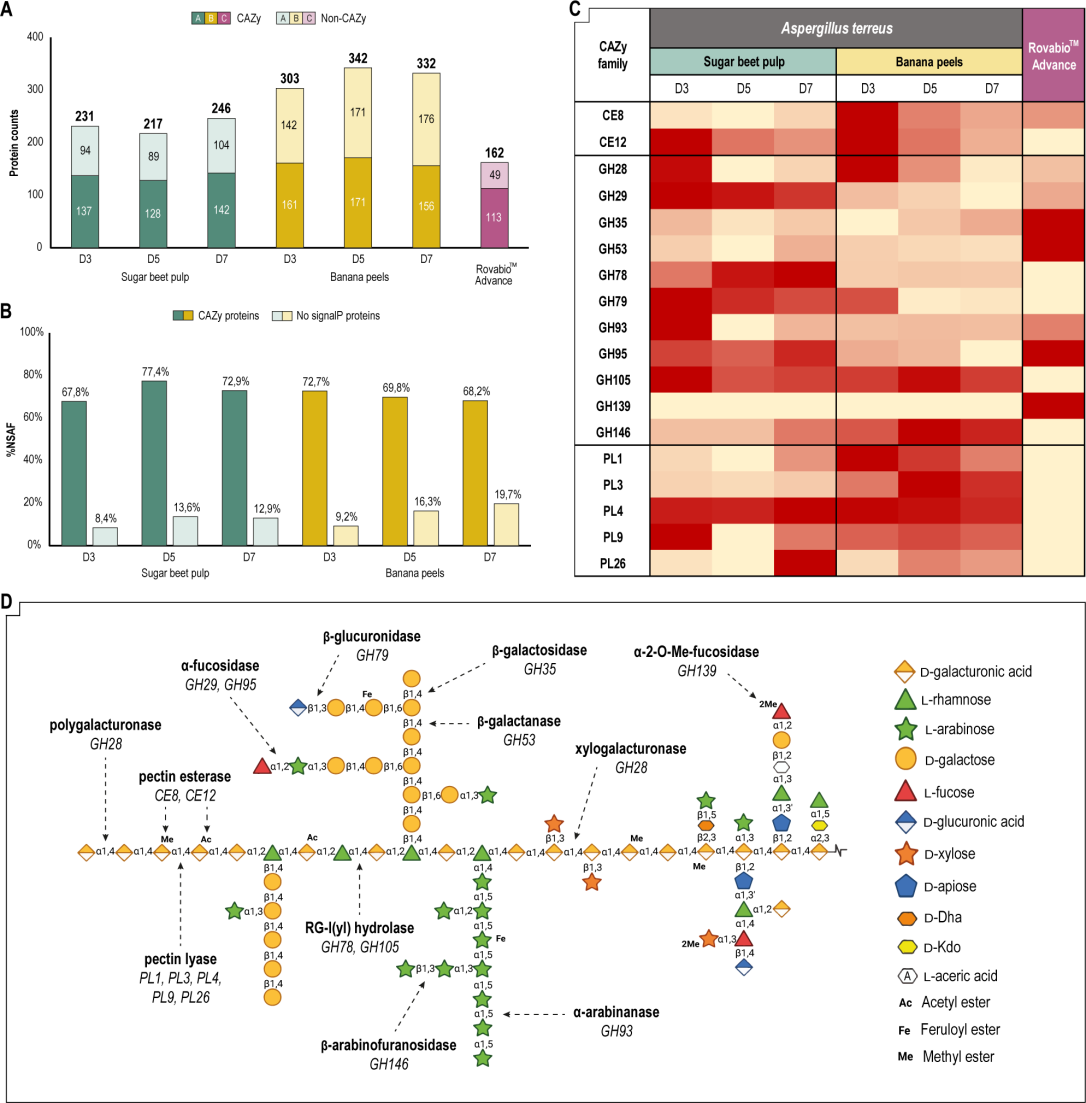
Proteomics analysis of *A. terreus* secretomes and Rovabio Advance. (**A**) Total protein counts according to CAZy annotation. (**B**) Abundances (% NSAF) of CAZy annotated proteins and proteins without SignalP prediction. (**C**) Absolute normalized quantities (NSAF) of selected CAZymes. Red indicates high quantity of the CAZy family in the sample. (**D**) Cleavage site of the CAZy families selected for their ability to degrade pectin. NSAF, normalized spectral abundance factor.

To better understand the enzymatic machinery behind Rovabio Advance and *A. terreus* secretomes, a shotgun proteomic analysis was performed focusing on CAZymes. Overall, the tandem mass spectrometry analyses revealed that *A. terreus* secretomes contained more proteins than Rovabio Advance (Fig. 4A), the maximum being reached with the secretome produced on banana peels at D5 with 342 proteins detected. When considering all secretomes, more than half of the secreted proteins were CAZymes, which is consistent with previous observations in ascomycetes secretomes (34). Interestingly, the *A. terreus* secretomes contained more CAZymes than Rovabio Advance, based on protein counts as proxy of their abundances (Fig. 4A). Moreover, banana peel secretomes displayed more CAZymes than the ones produced on SBP. Of note, in the banana peel secretomes, we observed a slight increase over time of proteins not predicted to be secreted (Fig. 4B). This observation could be explained either by a cell lysis during *A. terreus* culture on this substrate or by a misannotation of some of these proteins using SignalP. After careful analysis of the intracellular protein content in these secretomes, cell lysis was considered negligible (data not shown).

After validating the secretome integrity, we focused our attention on putative pectin-degrading enzymes (Fig. 4C). For this analysis, only CAZy families with previously reported activity on pectins (Fig. 4D) were selected (15). The abundances of pectin-degrading enzymes in the different secretomes were compared based on their normalized spectral abundance factor (NSAF) values, which normalize spectral counts of each protein per polypeptide length. The secretion of pectin-degrading enzymes by *A. terreus* was impacted by the inducer and the time of culture. For most of the CAZy families selected, maximal NSAF was reached at D3 of culture on both SBP and banana peels. Carbohydrate esterases (CEs) and polysaccharide lyases (PLs) were more secreted on banana peels than on SBP, while glycoside hydrolases (GHs) were more abundant in the secretomes produced on SBP. We also observed that polygalacturonases (GH28) and pectin acetyl esterases (CE12) were co-secreted by *A. terreus* on both inducers, which is in line with previous observations (35–37). Comparative analysis of *A. terreus* secretomes and Rovabio Advance revealed that many pectinolytic activities, i.e., CAZymes active on rhamnose (GH78) and unsaturated uronic acid-containing oligomers (GH105), as well as pectin, pectate, and RGI lyases from families PL3, PL4, PL9, and PL26, were absent from Rovabio Advance. Conversely, CAZymes from families GH35 (putative β-galactosidase), GH53 [putative β-(1,4)-galactanase], GH95 (putative α-fucosidase), and GH139 (putative α−2-*O*-Me-fucosidase) were more abundant in Rovabio Advance than in *A. terreus* secretomes.

### Matrix-driven correlation unveils key enzymatic activities involved in SBM degradation by fungal secretomes

**Fig 5:**
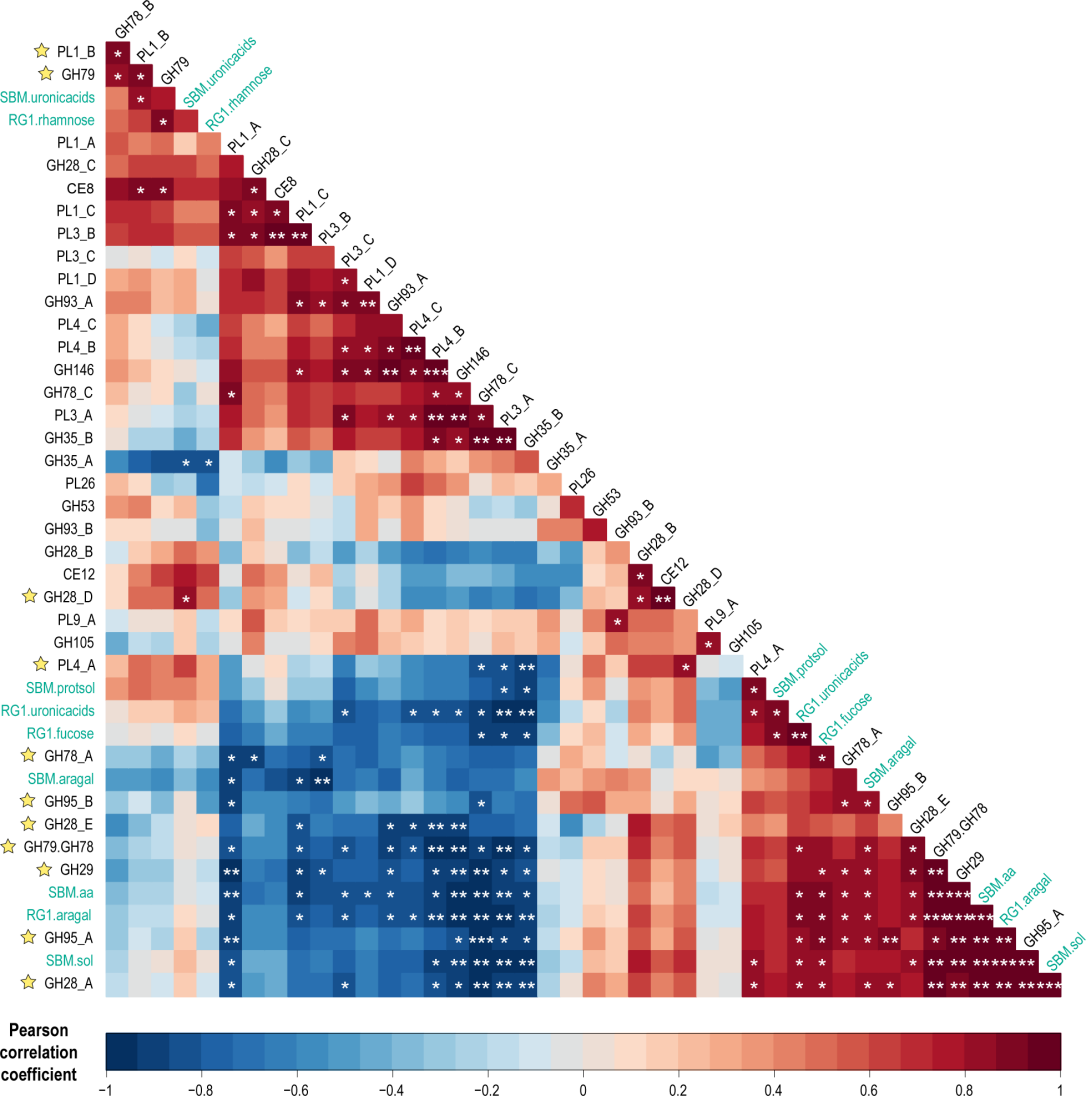
Pearson correlation matrix for hydrolysis performances and enzymatic composition of *A. terreus* secretomes. The markers SBM.fucose and SBM.rhamnose are not present as no fucose nor rhamnose was detected in the soluble fraction of SBM after hydrolysis by the fungal secretomes alone. *P < 0.05, **P < 0.01, ***P < 0.001. Enzymes having a significant positive correlation with at least one hydrolysis marker are identified with a yellow star. SBM.aa, release of free amino groups from SBM; SBM.protsol, solubilization of proteins from SBM; SBM.sol, solubilization of SBM; SBM/RG1.aragal, release of arabinose and galactose from SBM or RG-I; SBM/RG1.fucose, release of fucose from SBM or RG-I; SBM/RG1.rhamnose, release of rhamnose from SBM or RG-I; SBM/RG1.uronicacid, release of uronic acid from SBM or RG-I.

A correlation of proteomics data with the hydrolysis performances of secretomes was made using a Pearson correlation matrix (Fig. 5). The confidence level used for the statistical analysis was set at 0.95 to focus our interpretation on significant positive and negative correlations (*P* value of <0.05). As expected, the release of UA from SBM and RG-I is positively correlated with the presence in the secretomes of CAZymes cleaving the backbone of pectins (GH28_D and GH28_A, respectively). In the same trend, the release of fucose from RG-I is correlated with the presence of GH29 and GH95_A, which are both putative α-fucosidases. These observations validate our matrix-driven approach. Another interesting observation is that the hydrolysis of arabinose/galactose from the two substrates tested is significantly and positively correlated, potentially indicating structural similarities in RG-I side chains between SBM and commercial soybean RG-I. One of the highlights of the matrix presented in Fig. 5 was that all putative α-fucosidases of *A. terreus* were strongly positively correlated to the release of arabinose and galactose from SBM, suggesting that fucose hydrolysis is a key step to improve the degradation of pectin side chains, in line with the terminal position of fucose residues in RG-I (Fig. S3). Another observation was that among the five GH28 secreted by *A. terreus*, only two were positively correlated with the release of UA from soybean RG-I and SBM. This could either indicate that the three other GH28s are not targeting SBM pectins and soybean RG-I or that they display an endo-acting activity, releasing d-Gal*A* oligomers instead of monomers. In a similar way, among the 12 putative pectin lyases secreted by *A. terreus*, one PL1 and one PL4 are correlated with the hydrolysis of UA from SBM and soybean RG-I, respectively, while no pectin esterase could be significantly linked to any of the selected hydrolysis markers. However, the presence of a pectin methyl esterase (CE8) in the secretome was positively associated with the presence of pectin lyases of family 1 (PL1). The activity of fungal PL1 has previously been reported to be modulated by the methylation degree of pectin (38). Therefore, the concomitant prevalence of these two CAZyme families is relevant, considering their activity on methyl-esterified pectins. On the other hand, CE12 was positively correlated with two GH28s, suggesting these enzymes could be sensitive to pectin acetylation. Interestingly, a peculiar bimodular enzyme displaying GH78 and GH79 domains was positively correlated with the solubilization of SBM, the release of UA from RG-I, and the release of arabinose/galactose from both SBM and RG-I. This could imply that this bimodular enzyme contributes both to the hydrolysis of RG-I side chains by cleaving d-Glc*A*-β-(1,3)-d-Gal linkages with the GH79 domain and to the degradation of RG-I backbone by cleaving d-Gal*A*-α-(1,2)-l-Rha-α-(1,4)-d-Gal*A* linkages with the GH78 domain. In contrast, some enzymatic activities turned out to be negatively correlated with hydrolysis markers, suggesting that either they are not involved in or they exert an inhibitory effect on the degradation of pectins from SBM. As an example, PL3_A and GH35_B were negatively correlated with three markers of SBM hydrolysis and three markers of soybean RG-I hydrolysis.

## DISCUSSION

In a precedent study, we have demonstrated that *A. terreus* secretomes produced on SBP at D7 can increase the degradation of pectin in SBM by Rovabio Advance (22). In this study, we have deepened our analysis to unravel the enzymatic interplays involved in the fungal deconstruction of pectins by studying the synergy between *A. terreus* and Rovabio Advance for SBM degradation. We first confirmed that SBP and banana peels are both interesting culture inducers, promoting pectinase secretion by ascomycetes. The time-course analysis of *A. terreus* secretomes performed in the frame of this study revealed higher pectinolytic activity at the early stages of cultivation, in line with previous observations (39, 40). These results highlight the importance of the substrate inducer and the time of culture to fine-tune the fungal secretion of CAZymes (41, 42).

Fingerprinting SBM and RG-I hydrolysates with multiple hydrolysis markers resulted in a better understanding of pectins deconstruction by *A. terreus*. We showed that degradation of the pectin backbone and RG-I side chains by *A. terreus* secretomes is correlated with a higher release of small peptides and amino acids from SBM. This observation is consistent with a recent study demonstrating that most of the RG-I is covalently linked to arabinogalactan-proteins in the cell wall of *Arabidopsis* (13). In a different context, the action of the human colonic bacterium *Bacteroides thetaiotaomicron* on potato RG-I revealed that the deconstruction of pectin is orchestrated by several polysaccharide utilization loci (PULs), involving a huge variety of enzymatic activities (9). Transcriptomic analysis indicated the PUL involved in RG-I utilization by *B. thetaiotaomicron* exhibits genes encoding enzymes of families CE4, CE6, CE12, GH2, GH27, GH28, GH35, GH42, GH43, GH105, GH106, PL9, PL11, and PL26. As fungi do not possess PULs, we undertook a comparative secretomics approach. We revealed that the degradation of soybean RG-I is correlated with the presence of GH28, GH29, GH78, GH79, GH95, and PL4 in fungal secretomes. Interestingly, only polygalacturonases (GH28), α-l-rhamnosidases (GH78), and enzymes active on unsaturated oligogalacturonides (GH105) were highlighted in both studies, illustrating that the microbial enzymatic degradation of RG-I is a complex mechanism involving different CAZymes families, depending on the microorganism and the origin of RG-I. Previous correlations between fungal growth on pectins and pectin-degrading enzymes encoding genes also highlighted the intricacy of enzymatic machinery involved in fungal degradation of pectins (43).

The structure of RG-I can take multiple forms, depending on the plant origin and its processing (33). Although the molecular structure of soybean soluble polysaccharides was previously analyzed (32), the exact structure of SBM RG-I remains to be established. The harsh conditions applied to soybeans during the oil extraction process should impact RG-I structure and its interactions with other molecules. These structural differences were highlighted in our study through the comparison of commercial soybean RG-I hydrolysis and SBM hydrolysis by fungal secretomes. While *A. terreus* secretomes were able to release rhamnose from commercial RG-I (Fig. 3B), no rhamnose was detected in the soluble part of SBM after 48 h (Fig. 2B). In contrast, an additional effect of fungal secretomes was shown on Rovabio Advance for rhamnose release from SBM, but the opposite observation was made for commercial soybean RG-I. This paradox suggests that depending on the substrate tested, the RG-I-acting enzymes and rhamnosidases secreted by *A. terreus* differently catalyzed rhamnose residues and rhamnose-containing molecules.

Altogether, our results suggest the optimization path to enhance SBM hydrolysis by Rovabio Advance mainly lies in the degradation of the pectin backbone and of RG-I side-chain ends. Indeed, our study shows that the release of UA and fucose was considerably improved by the addition of *A. terreus* secretomes. To confirm these assumptions, it would have been interesting to complete our work by a time-course analysis of SBM and soybean RG-I degradation by *A. terreus* secretomes. Unfortunately, the small quantity of fungal secretomes available is a major bottleneck for more advanced investigations. Nevertheless, our observations are consistent with previous *in vitro* characterization of Rovabio Advance where the enzymatic cocktail had no effect on the release of UA from wheat, maize, and rice compared to the control (21). In contrast, the efficiency of Rovabio Advance to release arabinose and galactose from SBM can be explained by its high diversity of hemicellulases (44–46). Several *in vivo* studies have reported a synergy between cellulases, hemicellulases, and pectinases to improve feed digestibility and performances of broiler chickens (47–49). Beyond decreasing the amount of recalcitrant NSPs in feed within the animal gut, supplementing Rovabio Advance by *A. terreus* pectin-degrading enzymes could also release prebiotic pectin oligosaccharides (50–52), therefore expanding the potential of this study for animal intestinal health.

## MATERIALS AND METHODS

### Chemicals and reagents

All chemicals and reagents were purchased from Sigma-Aldrich (Burlington, MA, USA). Soybean RG-I was purchased from Megazyme (Bray, Ireland; supplier reference P-RHAGN). SBM and micronized SBP were provided by Adisseo France S.A.S. SBM is composed of a mix of five batches of soybean meals from different origins. Rovabio Advance was also provided by Adisseo France S.A.S. (batch no. 4864148576). The same batch of the liquid concentrated form, stabilized with 0.35% (wt/wt) of sodium benzoate, was used for all hydrolysis tests.

### Fungal strain and culture maintenance

The *Aspergillus terreus* strain CIRM-BRFM 111 was provided by the CIRM-CF collection (Centre International de Ressources Microbiennes–Champignons Filamenteux). The strain was isolated from soil (Loire-Atlantique, France) and authenticated by the CIRM-CF with molecular tools as previously described (24, 53). *Aspergillus terreus* CIRM-BRFM 111 was grown on two solid media: a minimum medium (malt extract 20 g/L, yeast extract 1 g/L, and agar 20 g/L) for sporulation and a potato dextrose agar medium (Becton Dickinson, Franklin Lakes, NJ, USA) for conservation.

### Preparation of the culture inducers

Secretion of pectin-degrading enzymes by *A. terreus* was induced by cultivating the strain on 30 µm micronized SBP and on banana peels. Banana peels (Cavendish variety, organic agriculture, origin Dominican Republic) were dried in a drying tunnel at 75°C for 4 h and crushed using a blender with rotating blades. Banana peels were then sieved, and only the particles smaller than 0.8 mm were used for the cultures. Both inducers were used at a final concentration of 15 g/L in the culture, as described in the following sections.

### Sugar composition analysis of plant fractions

To determine the monosaccharide content of soybean meal, sugar beet pulp, and banana peels, the NSPs were isolated by treating 100 mg of each sample in 1 mL 70% ethanol (vol/vol) in a boiling water bath for 30 min. After cooling, the solid was washed several times until the phenol-sulfuric acid test indicated no remaining sugar in the washing water. The samples were dried until stable weight was obtained, and approximately 5 mg of each was hydrolyzed in 12 M H_2_SO_4_ (Sigma-Aldrich) for 2 h at 25°C in a heating block. The molarity was diluted to 2 M H_2_SO_4_ after adding inositol as internal standard and subsequently heating for 2 h at 100°C. The individual neutral monosaccharides were then derivatized into alditol acetates. Liquid-gas chromatography (Clarus 580, Perkin-Elmer, Shelton, USA) was performed at 205°C with H_2_ as carrier gas to analyze the alditol acetate derivatives (54). The automated m-hydroxybiphenyl (55) was applied to determine Gal*A* and glucuronic acid content, combined and identified as UA content. Each monosaccharide content was expressed as the percentage of the ethanol insoluble material. Measures were performed in triplicate, and calibration was done using known concentrations of standard monosaccharide solutions.

### Production of fungal secretomes

Fungal secretomes were produced as previously described (22). Sporulation of *A. terreus* was performed by a 7-day cultivation on minimum medium at 30°C. Fungal spores were recovered with 10 mL of a sterile saline solution [0.9% (wt/wt) NaCl] containing Tween 80 and counted on a Thoma chamber. Cultures of *A. terreus* for secretome productions were performed in 500-mL Erlen baffled flasks containing 100 mL liquid medium. The culture medium is composed of 15 g/L (dry matter) of autoclaved induction substrate (i.e., micronized sugar beet pulp or dried and crushed banana peels) as carbon source, 20 g/L of maltose as starter, 1.8 g/L of diammonium tartrate, and 0.5 g/L yeast extract as nitrogen sources. The medium also contains a solution of salts (0.5 g/L MgSO_4_, 0.2 g/L KH_2_PO_4_, and 0.0132 g/L CaCl_2_). Cultures were inoculated at 2.10^5^ spores/mL and incubated at 30°C with orbital shaking at 120 rpm. Culture supernatants (i.e., secretomes) were harvested (total volume of 75–85 mL per flask) at days 3, 5, and 7 post inoculation. Two flasks per inducer and per day of cultivation were made. These biological replicates were treated separately for the whole process of secretome preparation. After harvest, the culture supernatants were sequentially filtered down to 0.45 µm polyethersulfone (PES; Merck-Millipore, Cork, Ireland) and stored at −20°C until further use. Secretomes were then defrosted and filtered at 0.22-µm PES (Merck-Millipore) before being concentrated and diafiltered using a 10-kDa cut-off PES membrane (Vivacell 100; Sartorius, Göttingen, Germany) against a sodium acetate buffer (50 mM, pH 5.2), to a final volume of 0.5–2.0 mL. Concentrated secretomes were finally aliquoted, flash-frozen in liquid nitrogen, and stored at −20°C until further use. The protein content of each secretome was analyzed by SDS-PAGE electrophoresis using Coomassie blue staining (Mini-PROTEAN TGX Stain-Free 10%; Bio-Rad, Hercules, CA, USA) and quantified by Bradford assay (Bio-Rad Protein Assay Dye Reagent Concentrate) (56, 57). Total protein quantification by the Bradford method was made using a bovine serum albumin standard range from 0 to 15 mg/L. As their SDS-PAGE profiles were comparable, the secretome replicates were pooled together.

### Enzymatic hydrolysis of soybean meal and soybean rhamnogalacturonan-I

Hydrolysis tests were performed as previously described (22). Reactions were carried out in 1 mL sodium acetate buffer (0.1 M, pH 4) at 37°C and 850 rpm for 48 h. Reactions were performed in 2-mL test tubes containing 150 mg of SBM or 50 mg soybean RG-I. *A. terreus* secretomes were tested at 0.3 mg of total proteins in the reaction, with and without Rovabio Advance. Rovabio Advance was loaded at 0.3 mg (for comparison with *A. terreu*s secretomes) and 0.9 mg (for the synergy tests with *A. terreus* secretomes) of total proteins in the reaction. Due to the small amount of secretomes available (especially secretomes collected at day 3), hydrolysis tests were performed only one time on SBM and on soybean RG-I. After hydrolysis, SBM and RG-I insoluble fractions were separated from the soluble fractions by centrifugation at 13,663 × *g* for 20 min at 4°C. Then, SBM pellets were washed twice in distilled water, and the insoluble residual dry matter was weighed after heating at 105°C for 48 h. The percentage of SBM residual matter was calculated as follows:


$$Insoluble\;residual\;matter\;\left(\%\right)=\;\frac{Mass\;of\;dry\;matter\;after\;hydrolysis\;\left(mg\right)\;}{Mass\;of\;dry\;matter\;before\;hydrolysis\;\left(mg\right)}.$$

The percentage of SBM solubilization by the secretomes was then determined as follows:


$$\text{SBM solubilization}\;(\%)=\%\;{\text{insoluble residual matter}}_{no\;enzyme}-\%\;{\text{insoluble residual matter}}_{\text{enzyme}}.$$

To stop the enzymatic reaction, SBM and RG-I soluble fractions were diluted by half in 0.2 M NaOH and filtered at 0.45 µm. Soluble fractions were stored temporarily at +4°C (maximum 5 days) or −20°C for longer storage to avoid sample degradation. *In vitro* digestibility of SBM was assessed by the quantification of total proteins in the soluble fraction with the Bradford assay (technical duplicates), as previously described (56, 57). Quantification was made using a bovine serum albumin standard (from 0 to 15 mg/L). The release of peptides and amino acids from SBM was quantified by the TNBS assay (technical duplicates). A semi-miniaturized protocol was adapted from previous descriptions (58, 59). Absorbance was read at 340 and 420 nm (60). Quantification was made using a l-leucine standard range (from 0 to 0.7 mg), and the final concentration of free amino groups in the soluble fractions of SBM was determined by averaging the concentrations obtained at both wavelengths. Pectin sugar monomers in the soluble fractions of SBM and soybean RG-I were quantified using enzymatic assay kits (Megazyme). The kit references K-URONIC, K-RHAMNOSE, K-FUCOSE, and K-ARGA were used for the quantification of d-glucuronic/d-galacturonic acids, l-rhamnose, l-fucose, and l-arabinose/d-galactose, respectively. Assays were performed in UV-star microplates (Thermo Fisher Scientific, Waltham, MA, USA) according to supplier recommendations.

### HPAEC-PAD analyses

The detection method is performed using HPAEC-PAD (DIONEX ICS6000 System, Thermo Fisher Scientific). The system is equipped with a CarboPac-PA1 guard column (2 × 50 mm) and a CarboPac-PA1 column (2 × 250 mm) kept at 30°C. Elution was carried out at a flow rate of 0.25 mL/min and 25 µL of samples was injected. The eluents used were 100 mM NaOH (eluent A) and NaOAc (1 M) in 100 mM NaOH (eluent B). The initial conditions were set to 100% eluent A, and the following gradient was applied: 0–10 min, 0%–10% B; 10–35 min, 10%–35% B (linear gradient); 35–40 min, 30%–100% B (curve 6); 40–41 min, 100%–0% B; 41–50 min, 100% A. Integration was performed using the Chromeleon (version 7.2.10) data software.

### Proteomic analysis of fungal secretomes

Proteins were denatured for 5 min at 99°C and then subjected to denaturing electrophoresis on NuPAGE gel for 5 min. The whole exoproteome contained in each sample was sliced as a single polyacrylamide gel band, treated, and proteolyzed with Trypsin Gold (Promega, Madison, WI, USA) in 50 mM NH_4_HCO_3_ as recommended (61). The resulting peptides were analyzed with a Q-Exactive HF (Thermo Fisher Scientific) tandem mass spectrometer coupled to an UltiMate 3000 Nano-LC System (Thermo Fisher Scientific) operated essentially as previously described (62). Briefly, 5 µL of acidified peptides were separated on a nanoscale PepMap 100 C18 nanoLC column (3 µm, 100 Å, 75 µm, i.d. ×50 cm; Thermo Fisher Scientific) at a flow rate of 200 nL/min using a 90-min linear 4%–25% gradient of 0.1% HCOOH/80% CH_3_CN against 0.1% HCOOH/100% H_2_O. Full-scan mass spectra were acquired from 350 to 1800 *m*/*z* in data-dependent acquisition mode with a Top 20 strategy selecting peptides with two or three positive charges for fragmentation with a dynamic exclusion time of 10 s, an AGC target of 1E5, and an isolation window of 1.6 *m*/*z*. Tandem mass spectrometry spectra were interpreted against the fungal annotated genomes (*Aspergillus terreus* CIRM-BRFM 111 or *Talaromyces versatilis* IMI 378536) with Mascot Server (version 2.6.1, Matrix Science). Peptides with *P* values below 0.05 were selected. Proteins were validated with at least two peptides at a false discovery rate (FDR) of 1%, but only proteins with at least three spectral counts were taken into further consideration. NSAF was measured for each protein and sample as the number of spectral counts per polypeptide divided by its molecular weight as proposed earlier (63). CAZymes and SignalP annotations of *A. terreus* CIRM-BRFM 111 and *T. versatilis* IMI 378536 proteomes were carried out by the CAZy team (AFMB, Marseille, France). Both molar quantities (NSAF) and relative abundances (% NSAF) of proteins were considered (64, 65).

### Statistical tests

One-way analysis of variance and post hoc Tukey’s honestly significant difference were performed on the hydrolysis data using an online calculator (statskingdom.com) at a significance level of 0.05. The correlation matrix was established with a Pearson statistical test at 0.95 confidence level, using the R-Studio software for Windows (version 2022.07.2; Posit Software, PBC, Boston, MA, USA). Calculation of the matrix was performed by the R Stats Package (version 4.2.2), while the visualization was done using the corrplot package (version 0.92). Hydrolysis data implemented in the matrix were the values obtained for SBM and soybean RG-I hydrolysis by the fungal secretomes alone. Proteomics data implemented in the matrix were the molar quantities (NSAF) of selected proteins in the fungal secretomes.

## SUPPLEMENTAL MATERIAL

Supplemental material aem.02153-23-s0001.docx Tables S1 and S2; Figures S1 to S4.

ASM does not own the copyrights to Supplemental Material that may be linked to, or accessed through, an article. The authors have granted ASM a non-exclusive, world-wide license to publish the Supplemental Material files. Please contact the corresponding author directly for reuse.
