# Supplementary material for: A time-course analysis of Aspergillus terreus secretomes reveals the importance of pectin-degrading enzymes to increase the digestibility of soybean meal
Source: Appl Environ Microbiol. 2024 Aug 20;90(9):e02153-23. doi: 10.1128/aem.02153-23 (PMC11409638; doi:10.1128/aem.02153-23)

**SUPPLEMENTAL MATERIAL**

**Table S1. Sugar content of soybean meal, banana peels and sugar beet pulp.**

The values of ethanol insoluble material (EIM) are expressed in percentage of total dry weight. The values of neutral sugars and uronic acids are expressed in percentage of the EIM. n.d.: not detected

**Table S2. Hydrolysis percentages of pectin monomers from soybean RG-I by *A. terreus* secretomes.**

Quantities were determined based on pectin monomers quantification in the soluble fraction of RG-I after 48h hydrolysis. The percentages of hydrolysis were calculated according to soybean RG-I sugar composition claimed by the supplier, based on an initial quantity material at 50 mg of RG-I.

**Figure S1. Qualitative and quantitative protein analysis of *A. terreus* secretomes.**

Panel (A): concentration of proteins in the supernatant of culture during the growth of A. terreus on sugar beet pulp and banana peels. Days of secretomes harvest are indicated (D3, D5, D7). Error bars correspond to the standard deviation for biological replicates (n = 2). Panel (B): SDS-Page profile analysis of A. terreus secretomes after concentration. Protein load: 15µg/well.

**Figure S2.** **Enzymatic hydrolysis of soybean meal by Rovabio™ Advance.**

Panel (A): percentage of SBM solubilization after hydrolysis. Panels (B to F): quantification of free amino groups (B), uronic acids (C), rhamnose (D), arabinose + galactose (E), and fucose (F) in SBM soluble fraction. Hydrolysis reactions were carried out at 37°C and pH 4. Rovabio™ Advance was tested at 0.3 (pink round marks) and 0.9 mg (red triangle marks) of total proteins. Values presented here are corrected by controls without enzyme and without substrate. For panels C to F, error bars correspond to standard deviations calculated from technical replicates (n = 3), while for panels A and B no replicates have been performed.

**Figure S3. Pectins structure and composition.**

Adapted from Plouhinec et al., 2023 (16). The presented figure is a generic representation of pectin, displaying the possible compositions and structures found in pectic polysaccharides. These structures may vary depending on plant origin. HG: homogalacturonan ; RG-I: rhamnogalacturonan-I ; RG-II: rhamnogalacturonan-II ; XG: xylogalacturonan.

**Figure S4.** **Quantification of uronic acids by HPAEC-PAD in the soluble fraction of RG-I after enzymatic hydrolysis.**

**Table S1**

|  | Soybean meal | | Banana peels | | Sugar beet pulp | |
| --- | --- | --- | --- | --- | --- | --- |
|  | Mean | *SD* | Mean | *SD* | Mean | *SD* |
| **% of dry weight** |  |  |  |  |  |  |
| Ethanol insoluble material (EIM) | 25.62 | *-* | 48.06 | *-* | 18.45 | *-* |
| **% of EIM** |  |  |  |  |  |  |
| Rhamnose | n.d. | *-* | n.d. | *-* | 1.38 | *0.11* |
| Fucose | 0.64 | *0.2* | n.d. | *-* | n.d. | *-* |
| Arabinose | 3.54 | *0.13* | 4.42 | *0.17* | 17.44 | *0.43* |
| Xylose | 2.04 | *0.46* | 4.58 | *0.30* | 1.83 | *0.24* |
| Mannose | n.d. | *-* | 2.75 | *0.38* | n.d. | *-* |
| Galactose | 5.65 | *0.60* | 2.28 | *0.6* | 5.15 | *0.79* |
| Glucose | 4.96 | *1.30* | 34.61 | *0.43* | 20.09 | *0.80* |
| Uronic acids | 3.45 | *0.30* | 12.94 | *1.54* | 19.43 | *1.93* |

**Table S2**


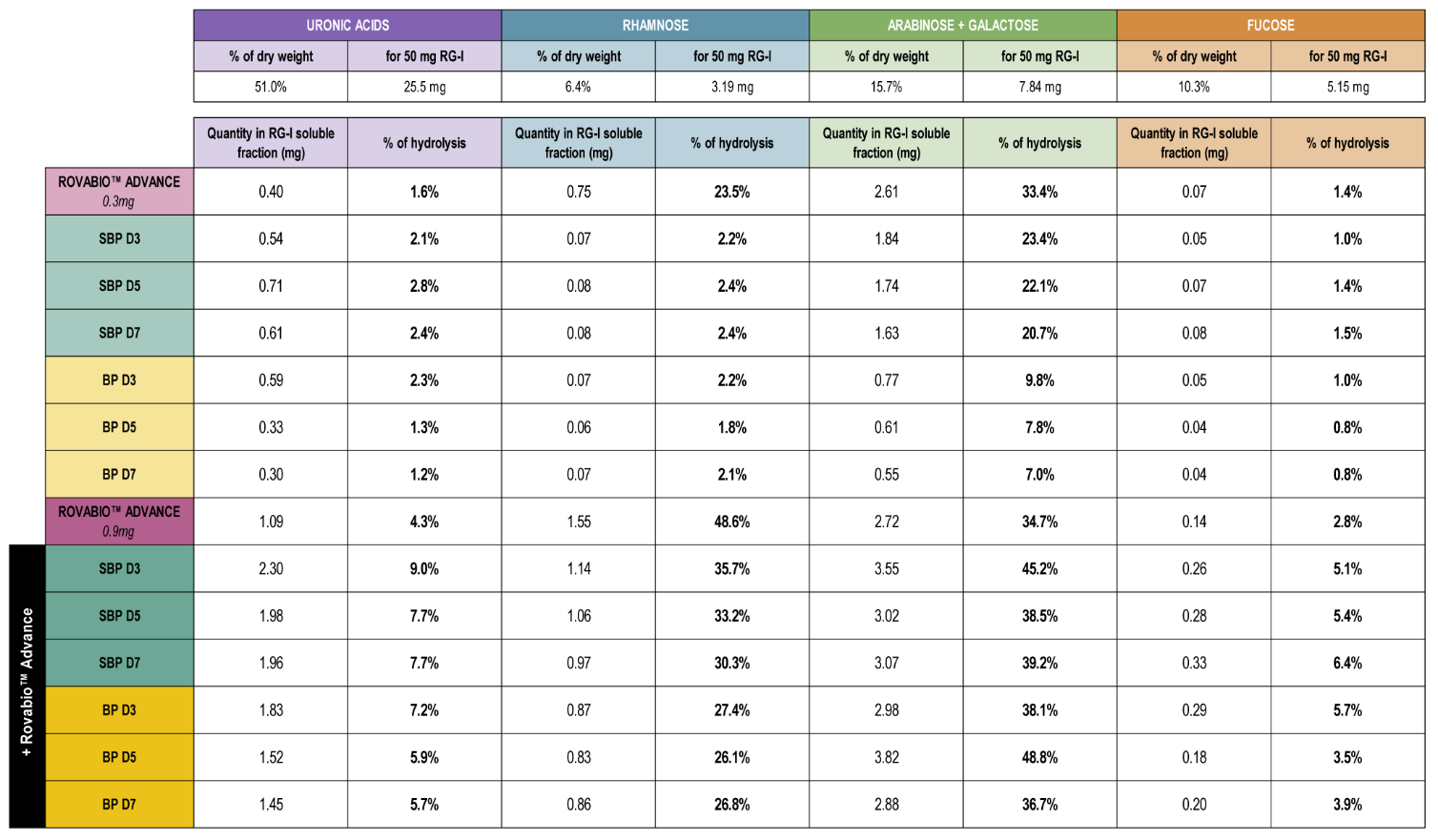


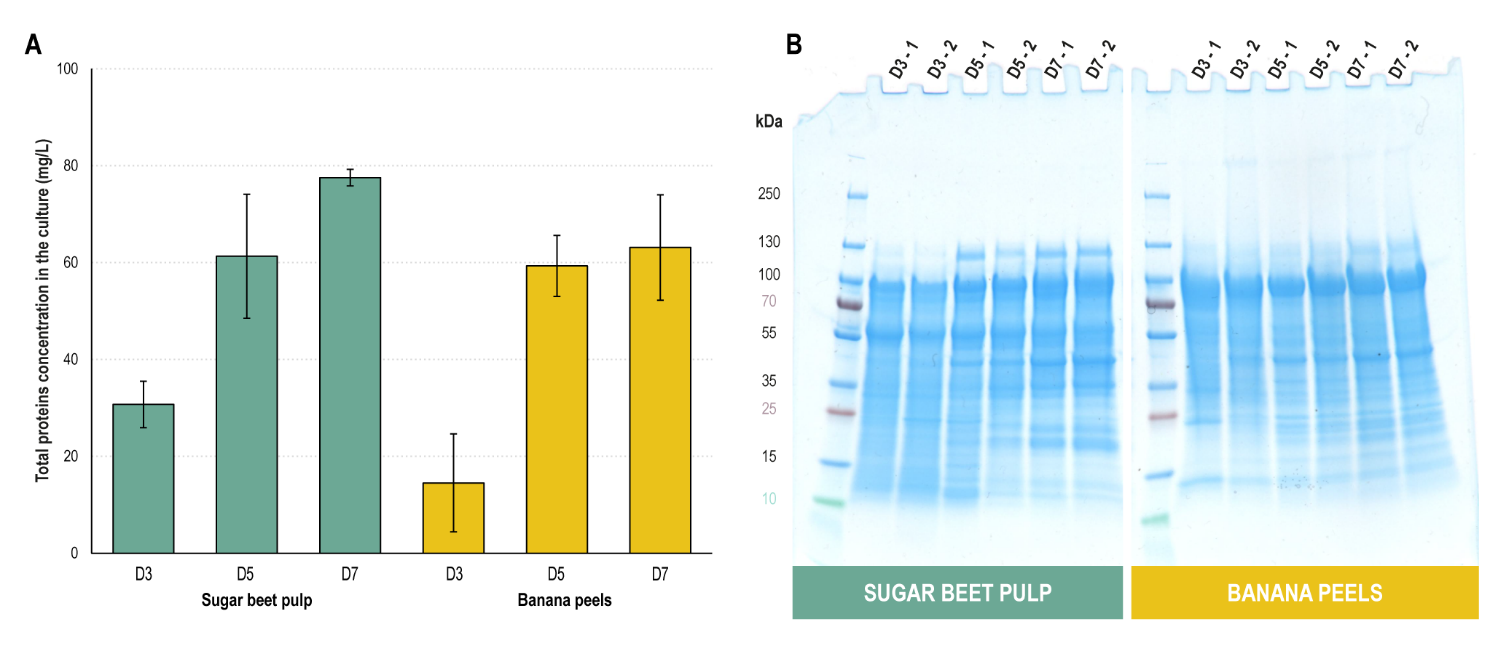
**Figure S1**

**Figure S2**


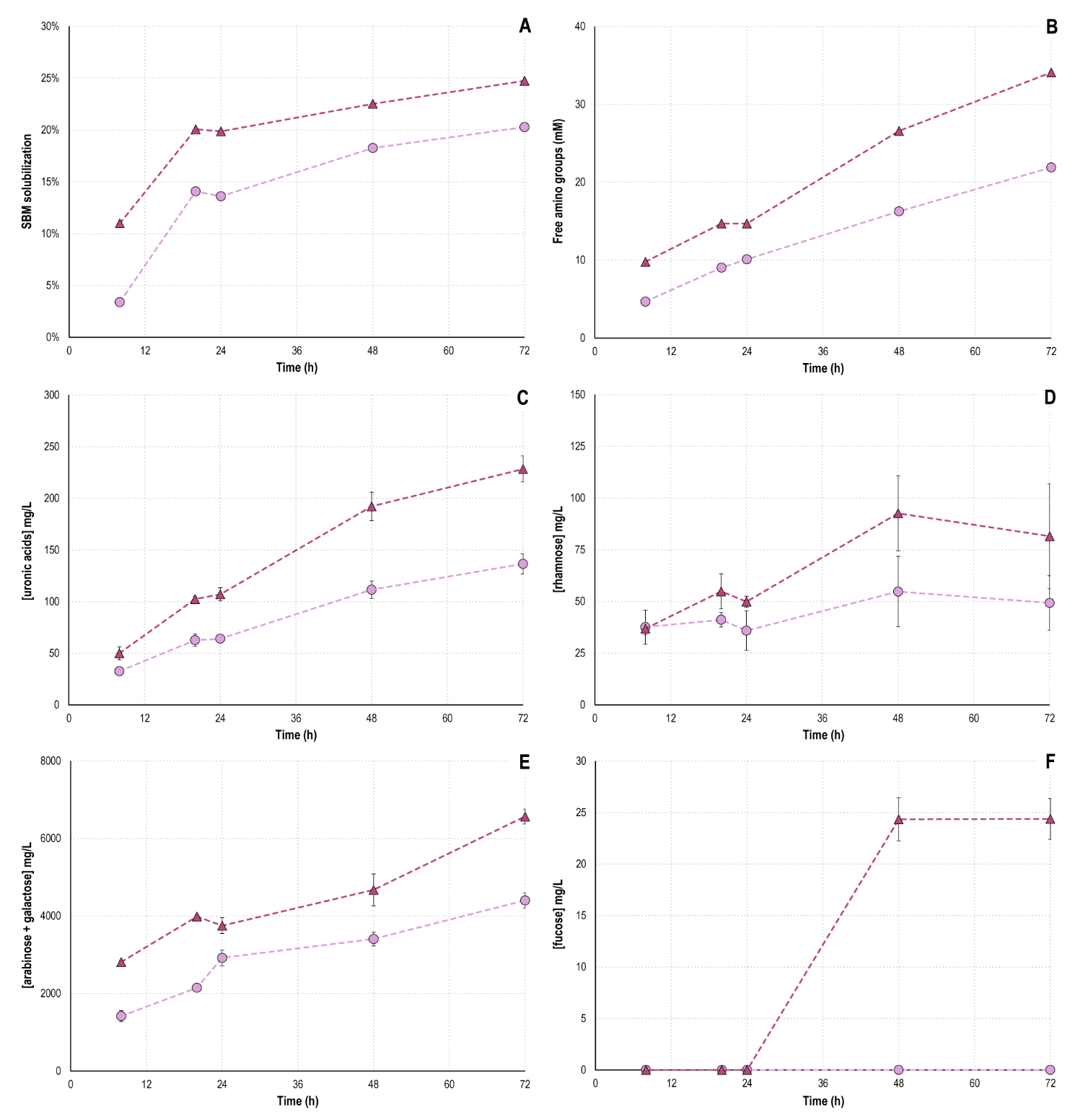


**Figure S3**


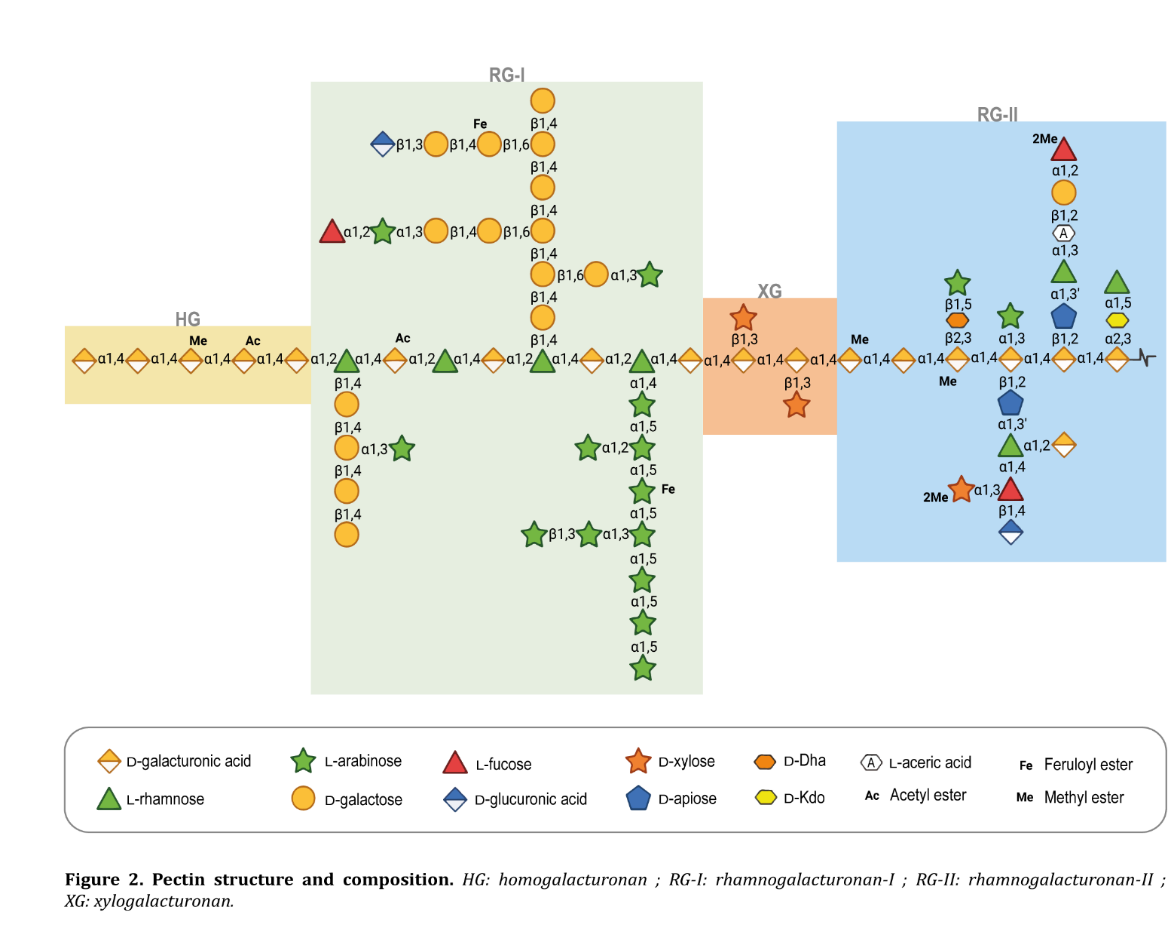


**Figure S4**


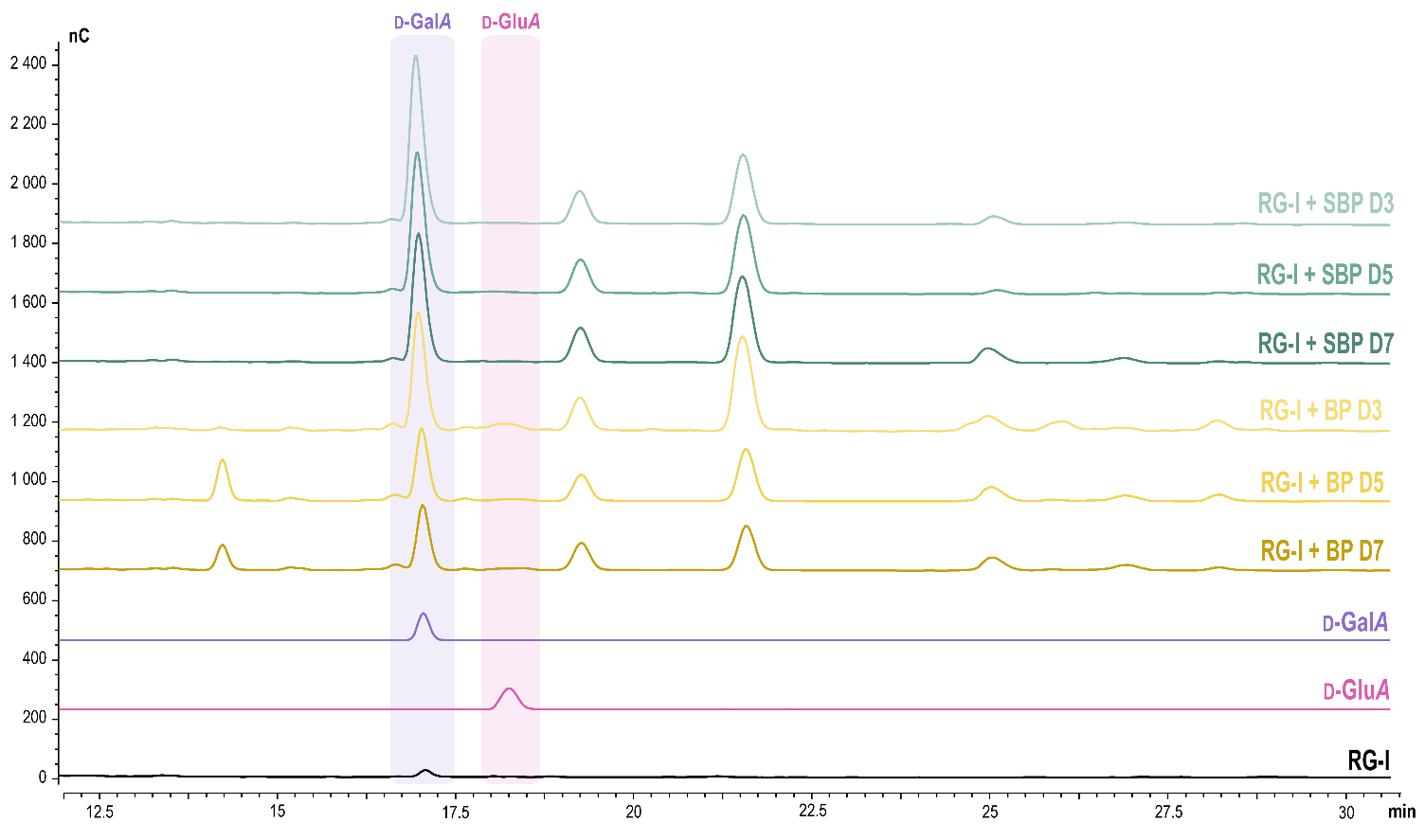

Supplement: Supplemental material — Tables S1 and S2; Figures S1 to S4. [file aem.02153-23-s0001.docx]
